# Engineering a Segmented Dual-Reservoir Polyurethane Intravaginal Ring for Simultaneous Prevention of HIV Transmission and Unwanted Pregnancy

**DOI:** 10.1371/journal.pone.0088509

**Published:** 2014-03-05

**Authors:** Justin T. Clark, Meredith R. Clark, Namdev B. Shelke, Todd J. Johnson, Eric M. Smith, Andrew K. Andreasen, Joel S. Nebeker, Judit Fabian, David R. Friend, Patrick F. Kiser

**Affiliations:** 1 Department of Bioengineering, University of Utah, Salt Lake City, Utah, United States of America; 2 CONRAD, Department of Obstetrics and Gynecology, Eastern Virginia Medical School, Arlington, Virginia, United States of America; 3 Department of Biomedical Engineering, Northwestern University, Evanston IL, United States of America; Burnet Institute, Australia

## Abstract

The HIV/AIDS pandemic and its impact on women prompt the investigation of prevention strategies to interrupt sexual transmission of HIV. Long-acting drug delivery systems that simultaneously protect womenfrom sexual transmission of HIV and unwanted pregnancy could be important tools in combating the pandemic. We describe the design, *in silico*, *in vitro* and *in vivo* evaluation of a dual-reservoir intravaginal ring that delivers the HIV-1 reverse transcriptase inhibitor tenofovir and the contraceptive levonorgestrel for 90 days. Two polyether urethanes with two different hard segment volume fractions were used to make coaxial extruded reservoir segments with a 100 µm thick rate controlling membrane and a diameter of 5.5 mm that contain 1.3 wt% levonorgestrel. A new mechanistic diffusion model accurately described the levonorgestrel burst release in early time points and pseudo-steady state behavior at later time points. As previously described, tenofovir was formulated as a glycerol paste and filled into a hydrophilic polyurethane, hollow tube reservoir that was melt-sealed by induction welding. These tenofovir-eluting segments and 2 cm long coaxially extruded levonorgestrel eluting segments were joined by induction welding to form rings that released an average of 7.5 mg tenofovir and 21 µg levonorgestrel per day *in vitro* for 90 days. Levonorgestrel segments placed intravaginally in rabbits resulted in sustained, dose-dependent levels of levonorgestrel in plasma and cervical tissue for 90 days. Polyurethane caps placed between segments successfully prevented diffusion of levonorgestrel into the tenofovir-releasing segment during storage.Hydrated rings endured between 152 N and 354 N tensile load before failure during uniaxial extension testing. In summary, this system represents a significant advance in vaginal drug delivery technology, and is the first in a new class of long-acting multipurpose prevention drug delivery systems.

## Introduction

The global HIV/AIDS pandemic continues to drive advances in biomedical technologies designed to quell the spread of the virus [1]. The recent USFDA approval of oral Truvada® for HIV pre-exposure prophylaxis (PrEP) in discordant couples is a major biomedical advance [2]. But clinicians have encountered difficulty in consistently demonstrating PrEP efficacy in prevention trials. In the CAPRISA 004 trial, topical vaginal application of the tenofovir (TFV) 1% gel used episodically before and after intercourse resulted in a 39% reduction in HIV infection in women [3]. But the same TFV gel did not reduce transmission rates when used once daily [4]. The variability in trial outcomes has caused consternation and several thoughtful reviews [5], [6], [7], [8], [9]. Yet the progress is evident when simultaneously considering the success of oral Truvada, the modest effect in CAPRISA 004, and alongside the accumulating evidence of the protective effect of TFV from non-human primate studies [10], [11], [12]. It is now conceptually clear that using oral and topical antiretroviral (ARV) drugs to interrupt the early events of sexual HIV transmission and dissemination is biologically and biomedically possible. The poor clinical outcomes result from confounding factors that span across behavioral, biological and pharmacological causes. Since the discovery of low rates of adherence in the Carraguard trial [13] and subsequently in the VOICE trial [4] it has been clear that infrequent use of gels by women likely has been a significant factor contributing to the low rates of effectiveness observed in most gel PrEP trials to date. For many reasons, trial participants are not sufficiently motivated to use the prevention products as instructed. Therefore, if PrEP is to be a technological success, new PrEP modalities are desperately needed that are easier to use and more desirable to women, and are supported by high user demand.

There are two main approaches being explored to increase user demand for PrEP, to either increase device duration or to make devices that are multifunctional and satisfy more than one user need. It is observed across many types of pharmaceutical products that user adherence and dose duration are positively correlated [14], [15]. To this end much effort has been directed toward long-acting injectable ARV [16] and ARV eluting intravaginal rings (IVR) [17], [18] for PrEP. Adding multiple functions, indications or purposes is also receiving attention as a method for improving user demand. Hormonal contraception is a well established technology in many low income countries impacted by the HIV pandemic. This has motivated the development of biomedical devices, and in particular long-acting IVR [19], [20], [21] that elute both contraceptive hormones and ARV. The development of multipurpose prevention technologies (MPT) could be ground breaking as there are no approved products that use two drugs to simultaneously address multiple indications. Yet the literature and the drug store shelf are replete with examples of fixed dose drug combinations designed to treat a single indication that were developed to improve ease of use, patient compliance and outcomes [22], [23].

As one might expect, there are a host of contraceptive agents and ARV under evaluation for use in MPT. The progestin levonorgestrel (LNG) is a leading contraceptive agent with a long history of clinical use in topical and oral administration. The Mirena® intrauterine system releases up to 20 µg LNG per day for 5 years. The WHO also developed a silicone reservoir IVR that released approximately 20 µg LNG per day for 90 days [24]. In a large multi-center clinical trial, the LNG IVR was over 95% effective in preventing unintended pregnancy. Tenofovir (TFV) is the leading ARV for many reasons including its approval for PrEP, its long clinical safety record (tenofovir prodrug formulations are currently used by 3.5 million people), extended duration of cellular levels following dosing and stability. But simultaneous dosing of TFV and LNG at relevant levels from simple, monolithic IVR is a challenge due to differences in their properties and target release rates, mandating the investigation of customized IVR designs.

We recently demonstrated the three-month zero-order delivery of TFV from a hydrophilic polyether urethane (HPEU) reservoir IVR [25]. In this report we describe the engineering and design of a segmented dual-reservoir IVR for the controlled delivery of TFV and LNG for 90 days. This manuscript illustrates the challenges of designing and integrating a solid, non-swellable LNG-releasing polyether urethane (PEU) reservoir into the existing swellable HPEU reservoir technology. We used a combination of *in silico*, *in vitro* and *in vivo* methodologies to study, engineer and evaluate the combination IVR. We present new insights regarding the use of the chemically diverse class of elastomeric PEU in reservoir-type dosage forms to deliver chemically diverse molecules at greatly divergent release rates. The work resulted in an IVR that gives nearly time-independent vaginal delivery of TFV and LNG for three months.

### Design

All clinically-used IVR in the U.S. employ reservoir technologies, however HIV prevention researchers have often investigated simpler matrix IVR designs to reduce manufacturing complexity and cost [26], [27], [28], [29]. Reservoir IVR are more attractive from a pharmacokinetic perspective as they can provide constant drug levels in the reproductive tract over extended durations [25]. Matrix IVR intrinsically provide high initial release rates, followed by continuously attenuated rates over time [17]. This is generally undesirable for PrEP applications, because the maintenance of preventative ARV levels for the intended IVR duration will require an excess of ARV exposure at early times during the release profile, presenting potential safety concerns. Furthermore, design limitations hindering the attainment of sufficient drug release rates can render it impossible to achieve high drug levels throughout the release profile and may result in increased probability of HIV acquisition late in the release profile of a matrix ARV-eluting IVR. These pharmacological limitations drive the investigation of reservoir IVR technologies that have the potential to provide precise and tunable control over the drug release rates for as long as several months.

**Figure 1:**
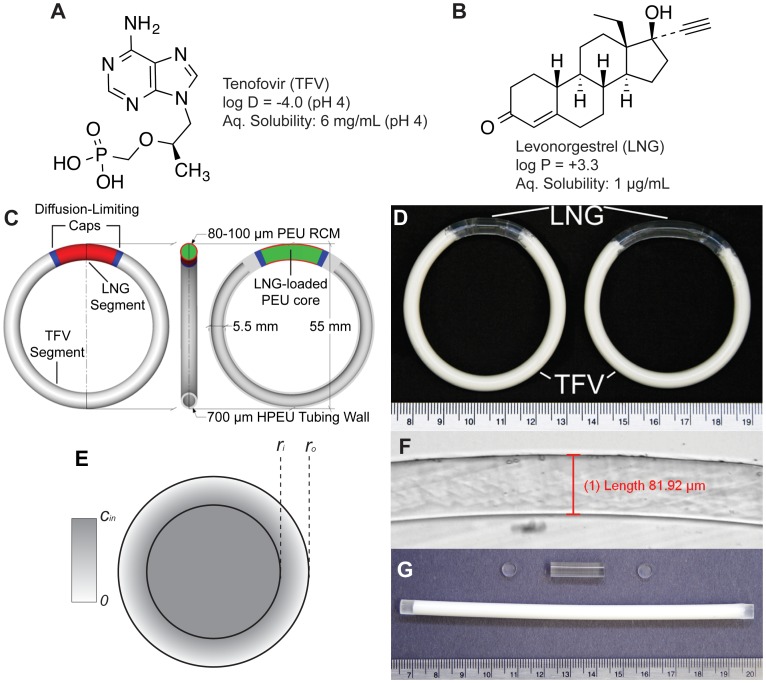
IVR Design Overview. Structural formulae of (A) the HIV-1 nucleotide reverse transcriptase inhibitor tenofovir (TFV) and (B) the progestin contraceptive levonorgestrel (LNG). (C) A design schematic of the full TFV/LNG IVR, shown in the 20 mm LNG segment configuration illustrating the LNG-loaded core (green), the rate-controlling membrane (RCM, red), diffusion-limiting end-caps (blue) and the hollow HPEU tube containing TFV-loaded paste (gray). (D) Photographs of TFV/LNG IVR in the 10 mm (left) and 20 mm (right) LNG segment configurations. (E) Illustration of a reservoir cross-section with outer and inner radii *r_o_* and *r_i_*, and core drug concentration *c_in_*. (F) Photomicrograph of the LNG segment cross-section showing microscopic measurement of RCM thickness. (G) Component parts of the TFV/LNG IVR: a TFV paste-filled HPEU tube (bottom), a co-axially extruded LNG-loaded reservoir segment (top) and two diffusion-limiting end-caps (left and right).

TFV and LNG exhibit approximately 7-log and 4-log differences in their partition/distribution coefficients and aqueous solubilities, respectively (Figure 1A and 1B), precluding their simultaneous solubilization and controlled release from a single polymeric compartment. To address these differences in molecular properties, we utilized chemically diverse polyether urethanes (PEU) capable of solubilizing and delivering TFV and other ARVs with varying physicochemical properties [30], [31], [32], [33]; an ability not generally afforded by the traditionally used silicone and poly(ethylene-co-vinyl-acetate) elastomers. Poly(ethylene oxide) can be added alongside the poly(tetramethylene oxide) soft segment typically used in PEU to create hydrophilic poly(ether urethanes) (HPEU) that swell in aqueous solution and can solubilize hydrophilic drugs when hydrated. Furthermore, PEU and HPEU are otherwise chemically identical and can be readily melt-welded to form mechanically sound IVR.

In addition to their disparate chemical properties, TFV and LNG also require vastly different release rates for their respective pharmacological effects. Our group designed an HPEU hollow-tube reservoir IVR to release 10 mg TFV per day [25], which we similarly targeted for the TFV/LNG IVR. However, as described above much lower release rates of LNG are employed for topical microdose contraception. We chose targets of 10 and 20 µg LNG per day, similar to the Mirena® intrauterine system and WHO's silicone LNG IVR. To address the challenge of delivering these diverse molecules at divergent release rates we designed a two-segment reservoir IVR system [31] containing an analogous TFV reservoir segment [25] and a PEU reservoir segment containing dissolved LNG. We desired to create a platform technology from which three devices, all with the same overall size (5.5 mm and 55 mm cross-sectional and outer diameters, respectively), could be fabricated to deliver ∼10 mg TFV per day with LNG daily doses of either 0 (TFV-only), 10 or 20 µg.

Generally, drug release rates (*dM/dt*) from end-capped cylindrical reservoir devices, like IVR or IVR segments can be varied by changing the segment length (*l*), core drug concentration (c*_in_*) or the outer, rate controlling membrane (RCM) thickness (represented by the subtraction of outer and inner cross-sectional radii, *r_o_−r_i_*). A steady-state description, assuming constant c*_in_*, is easily derived from Fick's laws and is ubiquitous in the design of reservoir IVR [34], [35]:
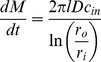
(1)where *D* is the effective diffusivity of the drug in the RCM material. To achieve the membrane-controlled release described in Equation 1, the drug-loaded core must be well-mixed so that the drug concentration remains uniform throughout the core and at the core/membrane boundary. For a solid polymer system, like the LNG segment, this is achieved by choosing a core polymer with much higher drug diffusivity than that of the RCM polymer. Specifically for PEU, drug flux can be hindered by increasing the volume fraction of crystalline hard segments. This is also easily maintained for a hollow-tube system like the TFV segment [25], because of the large differences in diffusivity between the liquid core and the porous membrane of the wall.

We chose to modify LNG release rates by changing the length of the LNG-segment. We desired to minimize LNG segment length to allow for minimal change in TFV dose between devices, but also sought to minimize the core LNG concentration because of potential physical stability issues of supersaturation and re-crystallization on the IVR surface [36]. Thus, we arrived at a target release rate 1 µg/mm/day for the LNG segment, leading to two IVR prototypes with segments of 10 and 20 mm length to deliver 10 µg and 20 µg LNG per day, respectively. A diagram of the 20 mm configuration is shown in Figure 1C, and a photograph showing both IVR configurations (with either 10 mm or 20 mm LNG segments) is shown in Figure 1D. We also added diffusion-limiting end-caps between the two segments (Figure 1C, shown in blue) to prevent circumferential diffusion of LNG from the solid PEU reservoir (green/red) to the HPEU tubing in the TFV-releasing segment (gray). The end-caps are composed of the same PEU used to create the RCM.

### Theory

Mathematical models of drug transport can aid the *in silico* design of drug delivery systems [37] including IVR [26], [38]. TFV release occurs at a constant rate as predicted by Equation 1, due to an excess of un-dissolved drug which maintains a constant TFV core concentration [25]. For the LNG segment in the TFV/LNG IVR, two issues confound the simple description of drug release presented in Equation 1. First, if LNG is equally distributed throughout the cross-section (core and membrane) at t = 0, then the device will initially behave as a matrix device, resulting in a period of burst release, which we refer to as the ‘burst regime’, prior to the onset of steady-state release [34]. Second, as LNG is completely dissolved in the PEU core, the drug source (c*_in_*) diminishes with time as drug is released, even after steady-state is achieved. We refer to this pseudo-steady-state (PSS) period as the ‘steady state regime’. We derived a comprehensive model which addresses both of these issues. Throughout, we assume the drug is completely dissolved at equal concentration in both the core and outer membrane at t = 0, and that equipartitioning exists between the core and RCM polymer. Axial diffusion is neglected because the loss of LNG to the end-caps occurs on a much longer time-scale than that of drug release.

#### Radial drug release from cylindrical matrices

Diffusion-limited drug release of a purely dissolved drug from a cylinder with no axial diffusion is presented in the literature [39]. At early times (up to ∼20–30% of cumulative release), the following first-term approximation predicts fractional cumulative release:
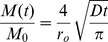
(2)where *M* is cumulative release, *M_0_* is the initial load of the device (or the cumulative release at infinite time), *D* is the effective drug-matrix diffusivity, and *r_o_* is the radius of the cylinder. Equation 2 will describe drug release in the burst regime for a solid reservoir with initial homogeneous drug concentration.

#### Computation of burst duration

We compute the burst regime duration assuming the device will proceed as a matrix until its initial steady state profile is reached. The steady-state profile is then integrated to obtain the mass loss required to complete the burst. First we solve Fick's Second Law in the steady-state form with a *c = c_0_* boundary at *r = r_i_* and a sink outer boundary (*c = 0* at *r = r_o_*) (depicted in Figure 1E):
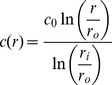
(3)We now integrate the right-hand-side of Equation 3 from *r_i_* to *r_o_* and scale appropriately to determine the mass remaining in the RCM at the beginning of the steady-state regime (*m_eq_*):
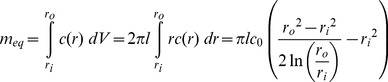
(4)To avoid confusion, in this manuscript a capital “*M*” will represent to the cumulative drug mass released from the device, while a lower-case “*m*” will represent the drug mass remaining in various compartments of the device. By subtraction we compute the total burst release (*M_b_*):
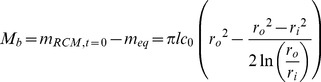
(5)Combining Equation 5 with Equation 2 and solving for *t*, we obtain the burst duration or length of the burst regime (*t_b_*):
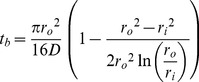
(6)There is some approximation in the computation of Equation 6, as concentration profiles in a matrix device following release of *M_b_* and in a reservoir system at steady-state are likely not identical. Some time is required for the system to adjust from the matrix to the reservoir profile when the system becomes constrained by the core boundary condition, during which additional drug is depleted from the core. Depending on the relationship between outer boundary flux during this transitional period and the predicted reservoir flux at when *c = c_0_*, Equation 11 (below) will over- or underestimate release rates during this period. This phenomenon is discussed further in the Supporting Information (see Table S1 and Figures S1 and S2 in File S1)

#### Pseudo-steady-state adjustment for variable core concentration

To effectively describe the attenuation of drug release rates in the steady-state regime, we employ a PSS technique which allows *c_in_* in Equation 1 to vary with time. First, we must replace *c_in_* with an expression which depends on the mass remaining in the device (which we denote as *m*) and the geometry of the device. However *m* must represent the total system drug mass (core and membrane):
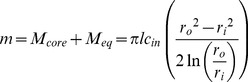
(7)Solving Equation 7 for *c_in_* and substituting into Equation 1 yields a simple ODE, although we must first replace *dM/dt*, which represents the rate of mass being released from the system, with the rate of mass loss from the system (*dm/dt*, equal to *−dM/dt*):
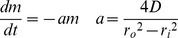
(8)Solving the ODE, applying the initial condition *c = c_0_* at *t_b_* gives an expression for the mass remaining in the steady-state-regime. Taking a time-derivative and applying the negative sign we obtain an expression for attenuated release rates as a function of time in the steady-state regime:
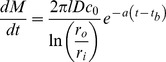
(9)
Equation 9 the can be integrated to obtain cumulative release in the steady-state regime:
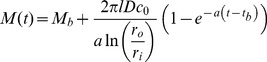
(10)We now have a simple exponential time constant (‘*a*’) to describe release rate attenuation. A PSS reservoir model was previously described for cylinders [40], but was complicated by consideration of axial release from the device, and did not consider the error in the depletion of drug from the outer membrane.

#### Complete two stage drug release model

We now have a complete, piece-wise description of cumulative drug release from the LNG segment, depending only on drug-polymer diffusivity in the RCM and device geometry:
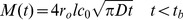
(11a)

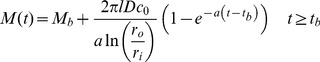
(11b)as described previously [26], daily release rates then are readily computed by subtraction. It should be noted that, as Equation 2 is a first-term approximation valid only at early times for a matrix device, Equation 11 will lose accuracy for proportionally thicker RCM (lower values of *r_i_/r_o_*). This is further discussed in the Supporting Information (File S1).

## Materials and Methods

### Materials

Aliphatic polyether urethanes Tecoflex EG-85A, Tecoflex EG-60D, Tecoflex EG-65D, Tecoflex EG-100A and the hydrophilic Tecophilic HP-100A-60 were purchased from Lubrizol Advanced Materials (Wickliffe, Ohio). Additionally, PEU-1 (73A-77A shore hardness), PEU-2 (59D shore hardness) and HPEU-35 (78A shore hardness, 37% equilibrium water absorption) were provided by DSM-PTG (now a subsidiary of DSM Biomedical, Berkeley, California). TFV was provided by Gilead Sciences (Foster City, California), and micronized LNG was obtained from Industriale Chemica (Saronno, Italy) or Haorui Pharma-Chem Inc. (Edison, New Jersey). USP grade glycerol was purchased from Spectrum Chemicals and Laboratory Products (New Brunswick, New Jersey). Solutol HS 15 was obtained from BASF (Florham Park, New Jersey). All water used was either USP grade or double de-ionized (DDI) through an 18 mΩ*cm filtration system. All other solvents were HPLC or ACS grade unless noted.

### LNG and TFV quantification by HPLC analysis

TFV and LNG quantification were performed using a 1200 Series HPLC (Agilent Technologies, Santa Clara, California) equipped with a diode array detector. TFV was quantified in *in vitro* release samples by a 15-minute gradient method described previously [25], [26]. Various HPLC methods were used to quantify LNG in unknown samples. In all cases a Zorbax ODS 4.6×250 mm, 5 µm column was used and LNG was quantified at 240 nm. For drug extraction samples a 5 minute isocratic run of 20∶80 (v/v) DDI H_2_O∶acetonitrile (ACN) was used, whereas a gradient method was used to quantify LNG in *in vitro* release samples. A longer gradient method was used to quantify LNG in samples containing both TFV and LNG due to interference from TFV elution. Both gradient methods are described in detail in the Supporting Information (see Tables S2 and S3 in File S1).

### Dissolution testing of matrix devices to determine LNG-PEU diffusivities

Drug-polymer effective diffusivity was determined using matrix-type PEU segments to aid in the design of the LNG segment and evaluation of RCM polymers. LNG was compounded by roll-coating and hot melt extrusion in each polymer using a Haake Minilab extruder (Thermo Scientific, Tewksbury, Massachusetts) to an approximate final diameter of 5 mm. Next, 2 mm length segments were excised from the extrudate batch and subjected to the extraction method below to determine the LNG loading for each batch. Segments approximately 15 mm in length were then cut and end-capped as described previously [27]. Before capping, the exact length and diameter of each segment were measured with digital calipers and the exact mass of each segment was measured with an analytical balance. Segments were subjected to release testing in sodium acetate buffer (pH 4.2) with 2% Solutol HS 15 [30] for 7 days at either 23°C, 37°C or 50°C. Aliquots of release media were collected daily and analyzed by HPLC. Daily release data were summed sequentially to determine cumulative release, which was normalized to the initial LNG mass in each segment. Linear regressions of cumulative data against the square-root-of-time were performed from day 3 to day 7 (some non-linearity was observed on days 1 and 2).

As discussed above, diffusion-controlled release from a cylindrical matrix device wherein the drug is completely dissolved and axial transport can be neglected can be approximated by a simple relation at early-times (Equation 2). If the expected linearity of fractional cumulative release (*M/M_0_*) against the square-root-of-time is observed experimentally, Equation 1 can be re-arranged to estimate the effective diffusivity:
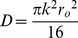
(12)where *r_o_* is the cross-sectional radius of the device and *k* is the slope of the linear regression of fractional cumulative release against the square-root-of-time.

### LNG Segment and IVR fabrication

A diagram detailing the TFV/LNG IVR manufacturing scheme is shown in the Supporting Information (Figure S3 in File S1). To fabricate LNG reservoir segments, LNG was first dissolved in either PEU-1 or EG-85A by hot-melt extrusion. PEU-1/EG-85A pellets were roll-coated with LNG powder (up to 2% w/w) and flood-fed into a KETSE 12/36 twin-screw extruder (TSE) (C.W. Brabender, South Hackensack, New Jersey) and cut using a Micropelletizer (Randcastle Extrusion Systems Inc., Cedar Grove, New Jersey). The pelletized extrudate was then subjected to extractions and HPLC analysis to determine drug content as described below. In some cases, LNG-loaded pellets were then mixed with placebo pellets to the desired average LNG loading (∼1.3% w/w). LNG-containing reservoir strands were then fabricated by co-axial extrusion. The TSE was connected to a 3/4″ single screw extruder (SSE) w/advanced torque rheometer drive (C.W. Brabender) at a 90° angle using a custom-designed mandrel crosshead (Guill Tool, West Warwick, Rhode Island). The LNG-loaded pellet mixture was starve-fed gravimetrically into the TSE using a KCL-24-KQx4 loss-in-weight feeder (K-Tron, Pitman, New Jersey) while PEU-2 or EG-65D pellets were flood fed into the SSE to form an outer rate controlling membrane (RCM) around the LNG-loaded PEU core. In some studies, EG-60D was used as an RCM polymer in place of EG-65D. The coated extrudate was passed through a water trough followed by cold air drying using an Air Wipe cooling ring connected to a Adjustable Spot Cooler vortexing tube (Exair, Cincinnati, Ohio) and fed to a conveyor belt, from which coaxial strands approximately 2–3 feet in length were manually cut. The feed rate of PEU-1, the SSE screw speed and the conveying speed were modulated to form a cylindrical reservoir extrudate with target dimensions of 5.5 mm outer diameter and 5.3 mm inner diameter (0.1 mm RCM thickness). In some experiments the RCM thickness was varied between 0.05 and 0.15 mm. To facilitate LNG diffusion into the RCM, thus mitigating the lag-times required to achieve steady-state release, strands were treated for 14 days at 40°C/75%RH prior to end-capping. In some cases heat treatment was varied between 4 and 76 days at 40°C/75%RH to assess the kinetic effects on lag/burst behavior during drug release. If segments were fabricated for inclusion in full TFV/LNG IVR, heat treatment was performed on the full IVR instead of the parent co-axial strand as described below. Segments of either 10 or 20 mm length were cut from their parent strands. A process flow diagram for LNG-loading co-axial extrusion is presented in the Supporting Information (Figure S4 in File S1). Exact lengths and diameters of LNG segments were measured by a digital caliper or thickness gage. Exact RCM thicknesses were determined by cutting thin slices of co-axial strand (either throughout the batch or proximal to individual samples) and imaging by bright field microscopy (Figure 1F). Four measurements per cross-section were taken using cellSens Standard software (Olympus, Center Valley, Pennsylvania) and averaged to determine a representative thickness for a given sample or batch. To prevent significant leakage of LNG into the TFV segment, LNG-loaded segments were end-capped with 2 mm length, 5.5 mm diameter EG-65D or PEU-2 caps using an HPS-EM induction welding system (PlasticWeld Systems, Inc., Newfane, NY). End-caps were cut from circular 5.5 mm EG-65D strands extruded using the SSE.

End-sealed TFV segments were fabricated by filling hollow hot-melt extruded HPEU-35 or HP-100A-60 tubes (5.5 mm outer diameter, 4.2 mm inner diameter, 145 or 155 mm length) with 2.1 to 2.3 g TFV/glycerol/water paste (62∶36∶2 w/w) and end-sealing by induction welding, as described previously [25]. Component parts of the TFV/LNG IVR (filled TFV segments, LNG segments and end-caps) are shown in Figure 1G. To form TFV/LNG IVR, capped LNG segments were welded to sealed TFV segments using a HPS-20 induction welding system with a split-die configuration (PlasticWeld Systems, Inc., Newfane, NY). TFV segments with 131 and 141 mm lengths (post-sealing) were paired with the capped 20 and 10 mm LNG segments, respectively, to form IVR with 55 mm outer ring diameter (Figure 1D). To form a more circular ring shape, IVR were annealed in custom-fabricated aluminum molds for approximately 15 minutes at 65°C. IVR were heat-treated for 14 days at 40°C/75%RH in sealed aluminum pouches prior to *in vitro* release testing.

Prior to any melt processing, all PEU or HPEU resins were dried overnight in a CAFM series compressed air dryer (Dri-Air, East Windsor, Connecticut) to less than 0.05% H_2_O (w/w) as determined using a C30 Coulometric Karl Fischer Titrator (Mettler-Toledo, Columbus, Ohio).

### Drug extractions to estimate LNG content and in vivo release

LNG was extracted from polymer segments to determine the LNG loading in each batch of co-axial extrudate. Several 2 mm long pieces were cut from various locations throughout the batch. Each piece was placed in a 5 mL volumetric flask, which was filled with 3 mL dichloromethane (DCM). Flasks were agitated on a High Capacity Mixer (Glas-Col, Terre Haute, Indiana) overnight to dissolve the PEU and LNG. Following dissolution, flasks were filled to volume with DCM. Flasks inverted several times to ensure good mixing. To precipitate PEU from the solution, 1.00 mL of the DCM solution was transferred to a 10 mL volumetric flask using a calibrated glass syringe. The 10 mL flask was then filled to volume with acetonitrile (ACN) and vortexed for 5 seconds. Aliquots of the resulting supernatants were then passed through a 0.2 µm PTFE syringe filter into HPLC vials for analysis. The same extraction procedure was also performed on 50 mg samples of LNG-loaded PEU-1/EG-85A pellets to assess the drug loading prior to co-axial extrusion.

The same procedure was also used to determine the average LNG mass loaded per device used in the rabbit PK study (see methods below), except that a 25 mL volumetric flask was used in place of a 5 mL flask for the first dissolution step. When full segments with end-caps were extracted, it was necessary to further cut the segment into 2–3 mm pieces before dissolution. These extractions were also performed after *in vivo* testing to estimate average LNG release rates.

### In vitro release testing of LNG segments and TFV/LNG IVR

LNG-containing PEU segments and TFV/LNG IVR were subjected to *in vitro* drug release testing. Samples were immersed in aqueous buffer sink for up to 90 days in 250 mL or 500 mL I-Chem glass jars (Thermo Scientific, Rockwood, Tennessee) in an incubated shaker cabinet set to 37°C and 80 rpm. Media were changed daily and media volumes were adjusted throughout the studies to ensure a sufficient sink for both TFV and LNG. TFV and LNG concentrations were not allowed to exceed 20% of their solubility in the release media. Typically, 25 mM sodium acetate buffer (pH 4.2) was used as the testing media, although Gibco (Life Technologies, Carlsbad California) 1× phosphate buffered saline (pH 7.4) was used for parallel *in vitro* release studies in the rabbit PK study to approximate the neutral vaginal pH typically observed in rabbits. Aliquots of release media were collected 23–25 hours from the previous media change and analyzed by HPLC at several points throughout each study to determine daily TFV and LNG release rates. To assess the validity of the model presented above, release rate profiles were compared to predictions from Equation 11. Some TFV/LNG IVR were also evaluate at an 200 rpm shaking speed to assess the adequacy of the standard 80 rpm in maintaining sink conditions. For *in vitro-in vivo* comparison, numerical integrations of *in vitro* release profiles were performed by trapezoidal approximation to generate time-averaged release rates.

### Evaluation of LNG containment by PEU end-caps

To evaluate the potential effectiveness of PEU end-caps in preventing LNG transport into the TFV segment, the full TFV/LNG IVR was rendered in COMSOL Multiphysics 4 and Fickian diffusion simulations were performed using the “Transport of Diluted Species” package. A diffusivity of 7.3×10^−11^ cm^2^/s was applied to the RCM and end-cap compartments based on an experimental measurement of LNG diffusivity in PEU-2. An LNG diffusivity of 3.0×10^−9^ cm^2^/s was applied to the core compartment and the tubing compartment of the TFV segment representing a typical softer PEU at 37°C (see Table 1), as the exact diffusivity in these compartments should not affect diffusion provided that the value is sufficiently high in the core for LNG to be well-mixed and sufficiently high in the tubing wall so that it acts as a sink. End-cap lengths of 1, 2 and 3 mm were evaluated, while the LNG segment length was fixed at 20 mm and the overall dimensions were fixed at 5.5×55 mm (cross-sectional and outer ring diameters). The RCM thickness was set to 100 µm, and was not loaded with LNG. An initial LNG loading of 42 mol/m^3^ (∼1.3%w/w, assuming a matrix density of 1.05 g/cm^3^), was applied to the core compartment. A free tetrahedral mesh was applied throughout the IVR, with higher node resolution in and near the RCM and near the segment/end-cap interface. Time-dependent effective diffusivity simulations, governed solely by Fick's 2nd Law, were carried out for approximately 5 years. Volume integrals of the end-cap and TFV segment compartments were performed every 7 days for the duration of the model and normalized to the initial mass of LNG loaded in the system.

**Table 1:** Measured LNG effective diffusivities for various PEUs, calculated using Equation 12. Data represents N between 3 and 8, mean ± SD.

Polymer	LNG Effective Diffusivity (cm^2^/s×10^−10^)
Tecoflex EG-85A	47±3
Tecoflex EG-100A	2.0±0.1
Tecoflex EG-60D	1.1±0.1
Tecoflex EG-65D	0.51±0.06
PEU-1	17±3
PEU-2	0.78±0.06

To experimentally validate the model presented above, TFV/LNG IVR (HPEU-35/PEU-1/PEU-2) were stored at 40°C for either 1, 3 or 6 months. IVR stored at −80°C were used as controls. IVR were dissected, and separate LNG extractions, as described above, were performed on the end-caps and TFV plug sections (first ∼5 mm of the TFV segment on either side of the LNG segment). As can be seen in Figure 1D, it is difficult to discern where the exact cap/segment interface is on the final IVR. Masses of the dissected LNG segments were compared to the initial segment mass before end-capping, and an adjustment was made to the LNG mass extracted from the end-caps based on the segment mass discrepancy and the initial LNG loading of the co-axial extrudate batch.

### Extension testing of TFV/LNG IVR

To assess mechanical robustness of the TFV/LNG IVR before and after 31 day *in vitro* release testing, we performed destructive extension tests to determine the tensile load and resulting extension required to cause joint failure. IVR were tested using an Instron 3342 with 500 N load cell and O-ring testing apparatus with Bluehill Lite software control (Instron, Norwood, Massachusetts). IVR were placed on the testing apparatus with the LNG segment oriented vertically and stretched at a rate of 5 mm/s until failure was observed, at which point the net extension and net load were recorded. The failure type of each IVR was also recorded.

### Pharmacokinetic testing of LNG-loaded segments in rabbits

Both 10 mm and 20 mm end-capped LNG-loaded PEU-1/PEU-2 reservoir segments were implanted into New Zealand white female rabbits (age 4–8 months) to assess *in vivo* device performance and LNG pharmacokinetics. This study was carried out in accordance with the U.S. Department of Agriculture's (USDA) Animal Welfare Act (9 CFR Parts 1, 2 and 3) and the Guide for the Care and Use of Laboratory Animals of the National Institutes of Health. The protocol was approved by the Institutional Animal Care and Use Committee of MPI Research (USDA Research License Number: 34-R-0031).

Preoperative and surgical procedures were performed as described previously [41]. Briefly, LNG-loaded segments were placed in the proximal, columnar epithelial region of the vagina, such that the distal end of the segment was approximately 7 to 12 cm from the introitus. Up to three 3-0 or 5-0 Prolene sutures were used to secure the segment to the outer ventral wall. All surgery was performed under analgesia and anesthesia, and all efforts were made to minimize suffering.

Animals weighed between 2.9 and 4.2 kg at implantation. Body weights of test subjects were recorded periodically during implantation. Groups of 6 animals were each administered either 10 or 20 mm segments, for either 28 or 90 days (4 groups total). In the 28-day groups, plasma was collected at 4 hours, 8 hours, and 1, 2, 3, 7, 14, 21 and 28 days post-implantation. In the 90-day groups, plasma was collected at 44, 60, 74 and 90 days post-implantation. Approximately 1 mL blood was drawn from the jugular vein of all animals at each time-point and stored in tubes containing tripotassium EDTA. Tubes were stored on wet ice until centrifuged under refrigeration, at which point samples were aliquoted into cryovials and stored at −50°C to −90°C until analysis could be performed. LNG was quantified in plasma samples by a validated LC-MS/MS method (described in the Supporting Information, see Tables S4 and S5 in File S1). To assess total LNG plasma exposure and device dose dependence, area under the curve (AUC) values were calculated from individual plasma data using WinNonlin Phoenix (Pharsight, St. Louis, Missouri). AUC was calculated from implantation to 28 days post-implantation (AUC_0–28_) for the 28 day groups and from 44 to 90 days post-implantation for the 90-day groups (AUC_44–90_).

All test subjects were euthanized at study's end (either day 28 or day 90) by an intravenous overdose of sodium pentobarbital solution followed by exsanguination. Approximately 500 mg of cervical tissue was collected from all animals during necropsy, snap frozen in liquid nitrogen and stored at −50°C to −90°C until LNG extractions could be performed. LNG was extracted from tissue and again quantified by a validated LC-MS/MS method (described in the Supporting Information, see Table S6 in File S1).

LNG segments were recovered post-study during necropsy. Time-averaged *in vivo* LNG release rates were estimated by subtracting the residual LNG mass recovered by extraction (method described above) from the estimated initial LNG mass loaded (batch average LNG loading multiplied by segment mass prior to end-capping), and dividing the net mass by the study duration. In addition, the average cumulative release values from the 28 day and 90 day groups were subtracted to determine the average release rate for the latter portion of the study.

## Results and Discussion

### LNG Diffusivity in PEUs

**Figure 2:**
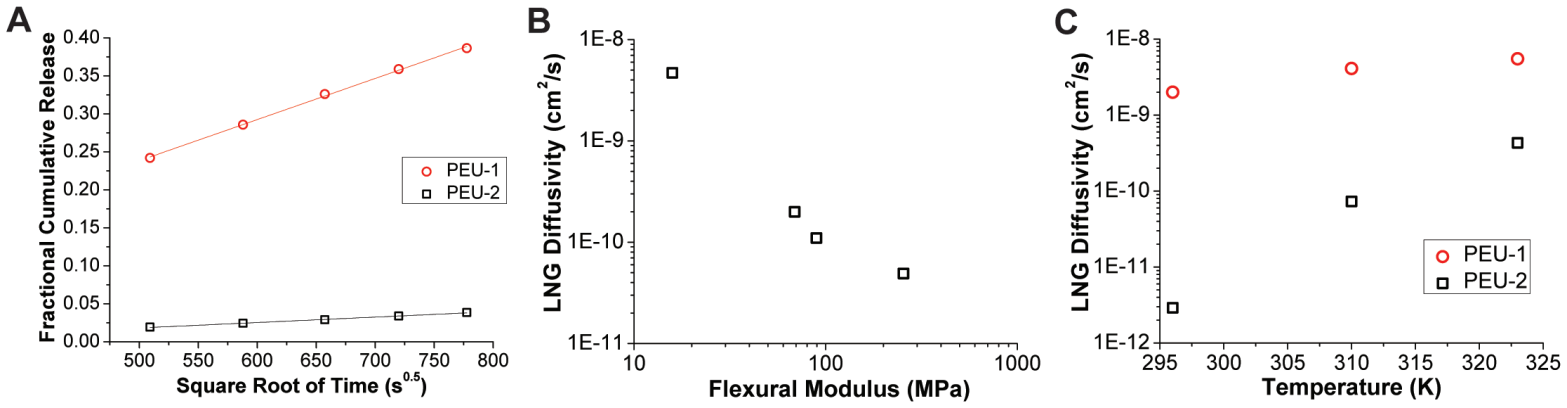
Determination of LNG effective diffusivities in PEU. (A) Example square-root-of-time fitting for PEU-1 and PEU-2 used to estimate LNG-PEU diffusivity values (Eqn. 12). (B) Log-log correlation between LNG diffusivity and reported flexural modulus for Tecoflex PEUs and (C) effect of temperature on LNG diffusivity in PEU-1 and PEU-2.

We measured effective diffusivities (*D*) for LNG in rate-controlling PEU by analyzing LNG release from cylindrical matrices and applying Equation 12. Figure 2A depicts examples of linear regressions of release against the square-root-of-time for PEU-1 and PEU-2 (R^2^>0.998). Diffusivity correlated negatively with manufacturer-reported flexural modulus for Tecoflex PEUs (Figure 2B). The mechanical properties of PEU can be controlled by modulating the molar ratio of the macrodiol (e.g. poly(tetramethylene oxide)) to the chain extender (typically a monomeric diol, e.g. 1,4-butanediol) [42]. Thus, the reduced diffusivity of a dissolved molecule, like LNG, in stiffer PEU, e.g. Tecoflex EG-60D and EG-65D, is likely a result of the lower volume fraction of the amorphous soft segment domains that allow LNG transport [43]. Figure 2C depicts the effect of temperature on LNG diffusivity. Interestingly, diffusivity increased 15-fold in the higher-modulus PEU-2 between 23°C to 50°C, but only 3-fold in PEU-1. This could result from differences in phase transition behavior of the crystalline hard segments between harder and softer PEU. Table 1 contains a summary of LNG diffusivities in various PEU tested. The lower diffusivity in PEU-2 at 23°C vs. 37°C necessitated heat treatment to allow LNG to load into the RCM, thus eliminating the undesirable lag time required to achieve steady-state release.

### Mathematical Modeling of LNG in vitro release

**Figure 3:**
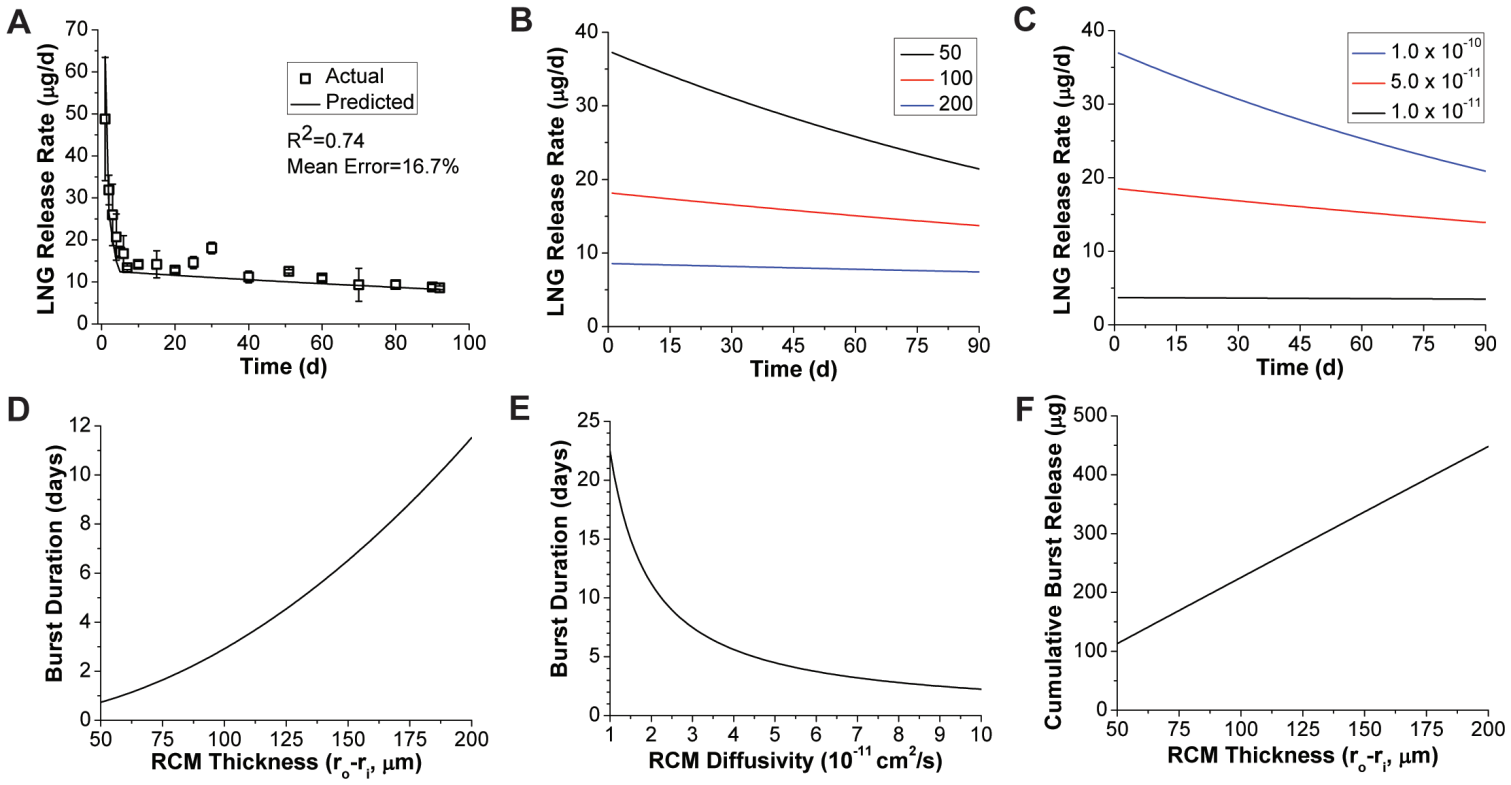
Model for dissolved drug release from cylindrical reservoirs. (A) Accuracy of the two stage drug release model (Eqn. 11) for LNG segments stored 76 days at 40°C to completely equilibrate LNG loading throughout the cross-section. Experimental data represent N = 3, mean ± SD. Effects of (B) EG-65D RCM thickness (in µm) and (C) diffusivity (in cm^2^/s) on the reservoir-stage model (Eqn. 11b) for 20 mm length segments with 5.5 mm diameter. Effects of (D) PEU-2 RCM thickness, and (E) diffusivity on the burst duration (*t_b_*) predicted by Equation 6. For panels C and E RCM thickness was fixed at 100 µm. (F) Maximal burst amount (*M_b_*) shown as a function of RCM thickness.

The model for drug release presented in Equation 11 proved useful in the design of the LNG reservoir segment by allowing accurate prediction of 90-day release rate profiles. Figure 3A depicts the full two-stage model (Eqn. 11) result in comparison to LNG release from PEU-1/PEU-2 segments stored at elevated temperature to homogenize LNG concentration across the device (as per the model assumption). The experimental *in vitro* release data agreed with the full two stage model (R^2^ = 0.74) with a mean prediction error of 17%. This correlation confirmed that our diffusivity measurements in rate-controlling PEU were sufficiently accurate for release rate prediction in the device design process, allowing us to screen potential RCM polymers for LNG diffusivity using simple cylindrical matrices produced on a small-scale batch extruder.

Use of the two-stage model has a practical limitation as the burst regime of the model is only accurate when complete RCM loading is achieved. Therefore, much of the remaining *in vitro* release data presented herein does not demonstrate the full burst predicted by the model. However, as demonstrated below the latter model regime was still useful regardless of the burst magnitude. The burst mass (*M_b_*) is then practically regarded as the maximal burst release that could be achieved from a given system.

Figures 3B and 3C depict the effects of RCM thickness and drug-polymer diffusivity on steady-state regime LNG release, predicted using Equation 11b, from 5.5 mm diameter solid reservoirs. As shown in Equation 8, the release rate time constant (*a*) varies with the ratio between diffusivity and RCM cross-sectional area. At a fixed diameter, diffusivity or RCM thickness are positively correlated with the magnitude of drug release, but also with the rate of drug release attenuation, as any increase in drug flux results in more rapid depletion of the core drug concentration. The burst duration (t_b_, Eqn. 6), is also shown as a function of RCM thickness and diffusivity in Figures 3D and 3E. The burst duration is inversely proportional to diffusivity, while the relationship between burst duration and RCM thickness is not clear from Equation 6 as *r_o_−r_i_* is not readily factored. As expected, the RCM thickness also correlates with *M_b_* (Figure 3F). Figures 3B–3F illustrate the complex relationship between burst duration, maximal burst mass and PSS release rate profile encountered when designing reservoir systems.

### In vitro release of LNG from end-capped segments

**Figure 4:**
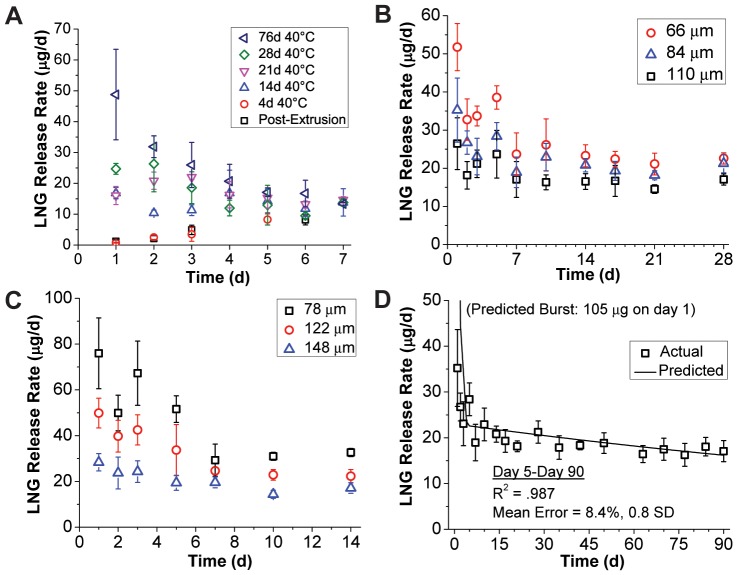
*In vitro* release of LNG from reservoir segments. (A) Effect of partial LNG loadings in the RCM on lag/burst behavior in LNG *in vitro* release kinetics for 10 mm length PEU-1/PEU-2 segments, achieved by 40°C storage for varying duration. *In vitro* LNG release from 20 mm length, 5.5 mm diameter co-axially extruded EG-85A segments with (B) EG-65D or (C) EG-60D RCMs of various thicknesses. (D) Full 90 day *in vitro* release kinetics from the 84 µm EG-65D RCM group with model comparison (Eqn. 11). All data represent N = 5, mean ± SD except panel A (N = 3). Segments in panels B–D were all stored for 14 days at 40°C before testing.

Experimentally, we evaluated the effects of several RCM design inputs on the LNG release profile, including RCM polymer selection, RCM thickness and duration of heat treatment required to load the RCM with LNG. First, effect of heat treatment was performed on segments fabricated from a LNG-loaded PEU-1 core and PEU-2 RCM by storing samples at 40°C for either 4, 14, 21, 28 and 76 days prior to *in vitro* release testing. As depicted in Figure 4A, a 14-day heat treatment mitigated both burst and lag behavior, and was thus incorporated into the manufacturing process. Furthermore, it was necessary to include a 14-day treatment at 40°C of the hydrophilic PEU used in the TFV segment (manuscript in preparation). This allowed us to reduce both procedures to a 14 day annealing of the final TFV/LNG IVR to both stabilize the TFV release rate on subsequent storage and eliminate the lag behavior in the LNG release profile [44].

Almost all reservoir devices will exhibit a drug burst that increases with time as drug is allowed to diffuse into the RCM. This phenomenon is acceptable if the amount of burst release does not result in unsafe drug levels. LNG is given orally as an emergency contraceptive at 1500 µg in a single dose, and our maximum predicted burst (*M_b_*) is over an order of magnitude lower that this dose as shown in Figure 4D. Furthermore, a range of release rates may be acceptable early in the release profile, provided that the eventually PSS release rate in the reservoir-stage is consistent and is consistently efficacious. If highly reproducible burst release profiles are required for other drugs, it may be necessary to manufacture devices with uniform concentration in both compartments to ensure a kinetically stable release profile during storage, either by compounding the drug in both polymers before co-axial extrusion, or by extended heat treatment. This was initially avoided to mitigate the potential for surface re-crystallization. However, in a recent stability study we discovered that LNG does not re-crystallize on the surface of the TFV/LNG IVR (to be reported in an upcoming manuscript).

Next, we evaluated the release of LNG from reservoir segments fabricated with an 1.3% (w/w) LNG-loaded EG-85A core and varying thickness of either EG-65D or EG-60D RCM (Figures 4B and 4C) (all samples heat-treated for 14 days at 40°C). Based on these data and the diffusion model, we selected an 80–85 µm thick EG-65D RCM to target 20 µg/day release on day 45 (the middle of the delivery profile), while also minimizing release rate attenuation throughout the reservoir-stage. The batch with the closest RCM (84 µm EG-65D) was tested for a full 90 days and compared to the model in the steady-state regime (Figure 4D). The reservoir-stage model (Equation 11b) and experimental data exhibited excellent correlation (R^2^ = 0.987 from day 5 to day 90), with mean error of 8.4%, or 0.8 experimental standard deviations.

### In vitro release of TFV and LNG from full two-segment IVR

**Figure 5:**
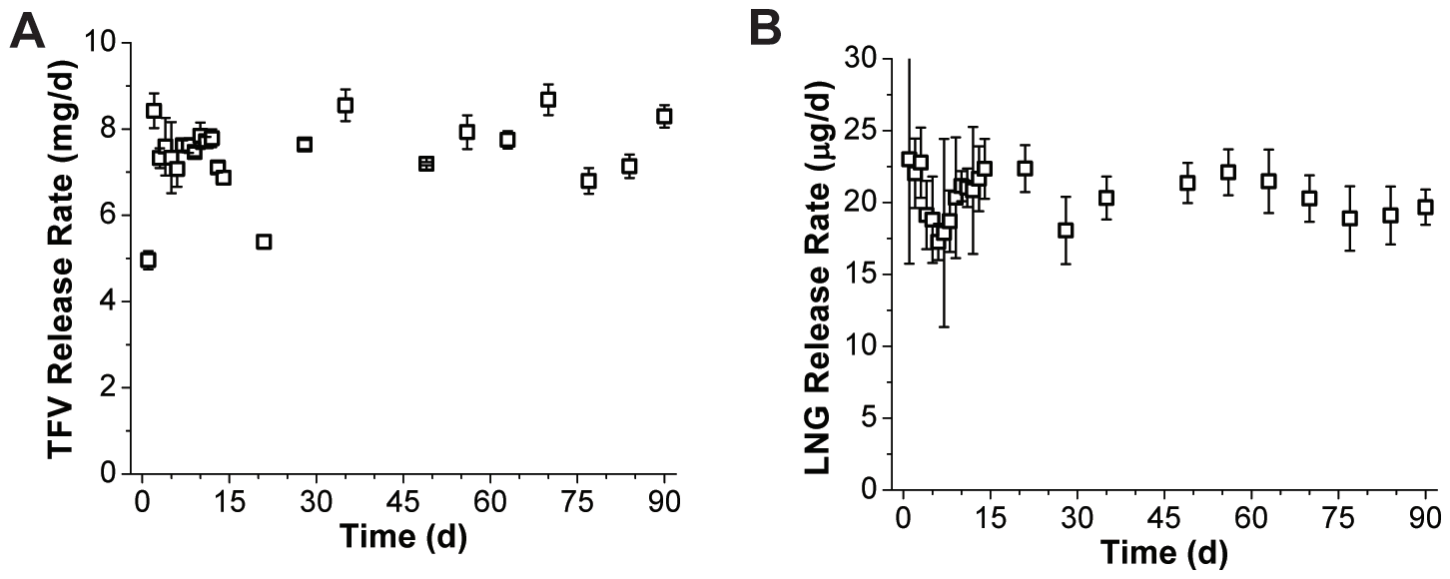
*In vitro* release of TFV and LNG from full two-segment IVR. *In vitro* release of (A) TFV and (B) LNG for 90 days from final TFV/LNG (20 mm) IVR prototypes using Tecophilic and Tecoflex polymers (HP-100A-60 TFV reservoir tube, EG-85A LNG reservoir core and EG-65D LNG reservoir RCM and end-caps). LNG reservoir RCM thicknesses were measured between 74 and 85 µm. All data represent N = 5, mean ± SD.

*In vitro* release of TFV and LNG from full IVR for 90 days is shown in Figures 5A and 5B. From day 2 to day 90, IVRs released TFV and LNG at near-constant rates of approximately 7.5±0.1 mg TFV and 21±2 µg LNG per day (N = 5 time-averaged IVRs, mean ± SD), a significant achievement given the disparity in drug properties and release rates.

Based on our recent sheep study comparing pharmacokinetics of the TFV-only IVR (similar *in vitro* release rate) and the TFV 1% gel [25], we hypothesize that a device with this constant TFV dose may protect women against HIV transmission. Also, based on previous data from the WHO silicone LNG IVR [24], we hypothesize that these LNG release rates can provide effective contraception. Given our understanding of how to predict release rates from this system, as well as the adaptability of the segmented IVR design, the release rates of TFV and LNG could be modified should human PK testing suggest different doses are needed.

TFV/LNG IVR evaluated at an increased shaking speed of 200 rpm released 23±1 µg LNG per day over 14 days (N = 3 time-averaged IVRs, mean ± SD), compared to 20±2 µg LNG per day at 80 rpm (N = 5). This comparison suggests that an 80 rpm shake speed sufficiently disrupts the unstirred layer, resulting in sink conditions appropriate for the model presented in Equation 11.

### Prevention of LNG diffusion between segments with PEU end-caps

**Figure 6:**
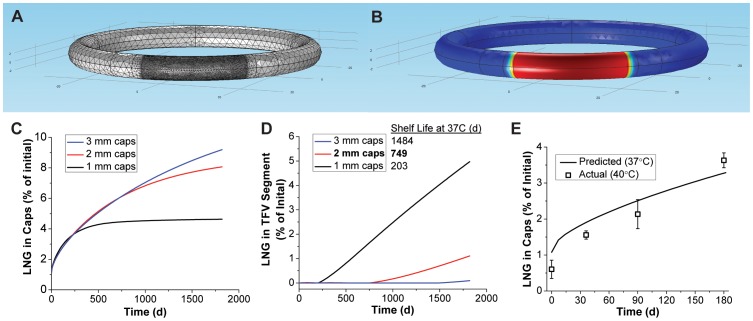
Prevention of LNG diffusion between segments by PEU end-caps. An example (A) finite-element mesh and (B) outer surface model result from the IVR storage diffusion model. The model result represents approximately 2 years storage at 37°C for an IVR with 2 mm length PEU-2 end-caps, where red represents maximum scaled LNG concentration, and blue represents zero LNG. Quantitative results show LNG leakage into the (C) end-caps and (D) entire hollow-tube in the TFV segment during storage by volume integrals of the COMSOL model. Shelf life was determined as the last time-point of complete LNG containment (none detected in the TFV segment). (E) Experimental determination of LNG loss into the caps as a function of TFV/LNG IVR (PEU-1/PEU-2 LNG segments) storage time at 40°C and comparison to the COMSOL result for 37°C storage.

One of the design challenges with developing multi-segment drug delivery systems is the potential for drug diffusion between compartments during storage of the drug product. While diffusion of TFV from the hollow core compartment is hindered by its poor solubility in HPEU [26], LNG diffuses effectively through PEU and thus its “leakage” into the TFV segment is expected. Therefore, we designed end-caps to prevent LNG diffusion into the TFV segment to maintain both LNG release rate magnitude and control in the TFV/LNG IVR following product storage. We evaluated the feasibility of using end-caps made from the same polymers as the LNG-segment RCM (PEU-2 and 65D), but aimed to minimize the end-cap length due to their mechanical rigidity (EG-65D has a flexural modulus of 255 MPa) and the fact that a large cap would eliminate additional length from the TFV segment. We modeled the complex multi-compartment nature of the problem by rendering a full TFV/LNG IVR performing finite-element simulations of Fickian diffusion (Figures 6A–B). Simulations were carried out for 5 years at 37°C, rather than the usual 40°C, due to availability of drug-polymer diffusivities at 37°C from studies presented above. Volume integrals were performed in both the end-cap and TFV segment compartments and normalized to the initial drug load in the 20 mm LNG segment. As depicted in Figures 6C and 6D, the 1 mm, 2 mm and 3 mm end-cap simulations predicted full LNG containment (no LNG in the TFV segment) for 203, 749 and 1484 days at 37°C, respectively. The 2 mm cap configuration ultimately was chosen because complete LNG containment was achieved in the model for 2 years at 37°C, at which time only 6% of the total LNG load had escaped into the end-caps. In Figure 6D, we show that the subsequent leakage into the whole TFV segment, proceeds at a linear but minimal rate (approximately 1% of total LNG load per year for 2 mm caps), suggesting that performance and chemical stability (depending on the quantitative metric used) may be achieved for much longer than 2 years in the 2 mm configuration. The end-caps also function as RCM, where thickness can modulate LNG flux at the TFV/LNG segment interface through the concentration gradient across the segment. Encouragingly, EG-65D (used in our current lead design) exhibits even lower LNG diffusivity than PEU-2 (Table 1), likely lengthening complete containment times from those observed in these simulations.

In an attempt to experimentally approximate the diffusion simulations, TFV/LNG IVR were stored for 6 months at 40°C and the caps and TFV segment plugs were subjected to LNG extractions at various times. The results are shown in Figure 6E. Reasonable agreement was observed between the theoretical and experimental LNG content in the caps, even with the temperature disparity (37°C to 40°C from model to experiment). Trace amounts of LNG (<5 µg total or <0.1% of total load) were detected in the TFV segment plugs, but this likely was not due to leakage through the end-caps as no increase was observed in LNG levels over time.

An “ideal” multi-segment IVR would be thermodynamically stabilized by caps formed from a material miscible with the polymers used and impermeable to all components in the formulation. Here we have demonstrated a practical, kinetically-stabilized system using a material already present in the IVR.

### IVR extension testing

**Figure 7:**
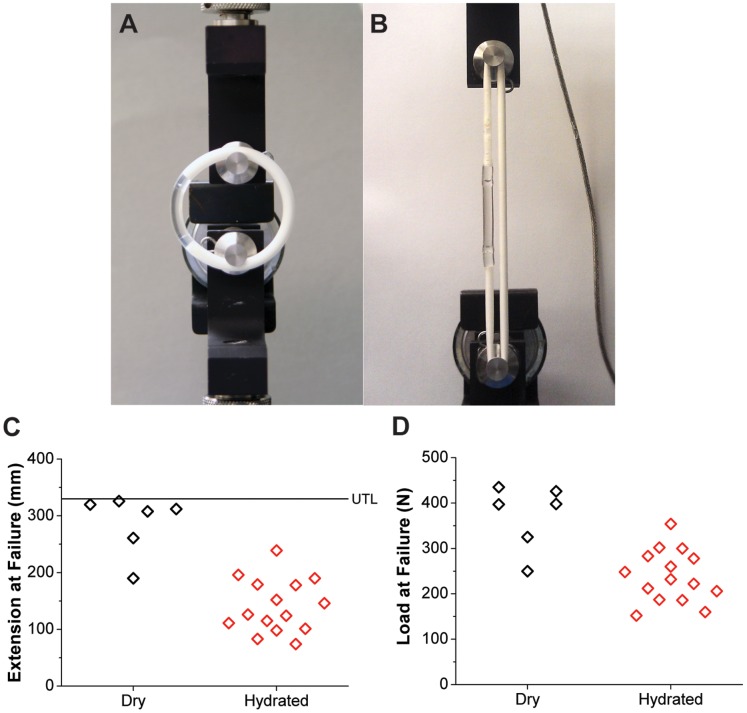
IVR extension testing. (A,B) Extension testing of a full TFV/LNG IVR to 101 mm extension and 145 N (32.6 lbf) load. (C) Extension and (D) load at TFV/LNG IVR both before (“dry”) and after (“hydrated”) 31 day *in vitro* release testing (N = 15 IVR per group). Nine of the 15 IVR in the dry group did not fail below the upper test limit (UTL, ∼330 mm extension).

Mechanical integrity is an important design consideration for any IVR. In this particular IVR design, we were faced with the additional challenge of welding swellable HPEU (TFV segment) to rigid, non-swellable PEU (capped LNG segments) while maintaining mechanical integrity both in dry and hydrated ring states. To evaluate this, we performed O-ring extension tests (Figures 7A and 7B) on full IVR after production (dry) and subsequent 31 day *in vitro* release testing (hydrated). As shown in Figures 7C and 7D, all IVR (N = 15) in the dry group endured at least 190 mm extension and 250 N (56.2 lbf) load before failure, and 9 of the 15 rings remained intact following extension to the limit of our instrument (approximately 330 mm deformation). IVR in the wet group exhibited extension and tensile load of 141±47 mm and 239±58 N (N = 15, mean ± SD) at failure, with all IVRs enduring at least 74 mm extension and 152 N (34.2 lbf) tensile load before failure. Thus, a reduction in joint integrity was observed when IVR were soaked in aqueous media, however these data demonstrate that the IVR are still mechanically sound and would require intentional and excessive user-applied stress to separate the segments. In the dry group, all observed failures (6 of 15) occurred between the cap and the LNG segment, whereas, in the hydrated group, an equal number of joint failures occurred between the cap and the LNG solid reservoir versus the cap and the TFV tube plug (7 of each). The remaining hydrated ring experienced a HPEU tubing rupture near the tube seal, but at the highest observed load at failure in the hydrated group (354 N/79.6 lbf). Given the mismatch in the HPEU tube, which exhibits 58% equilibrium swelling by mass, and the non-swellable PEU assembly (caps and LNG reservoir), the mechanical joint integrity observed in dry and hydrated IVR is remarkable and illustrates the utility of the PEU/HPEU platform in sophisticated drug delivery systems.

### PK testing of the LNG segment in rabbits

**Figure 8:**
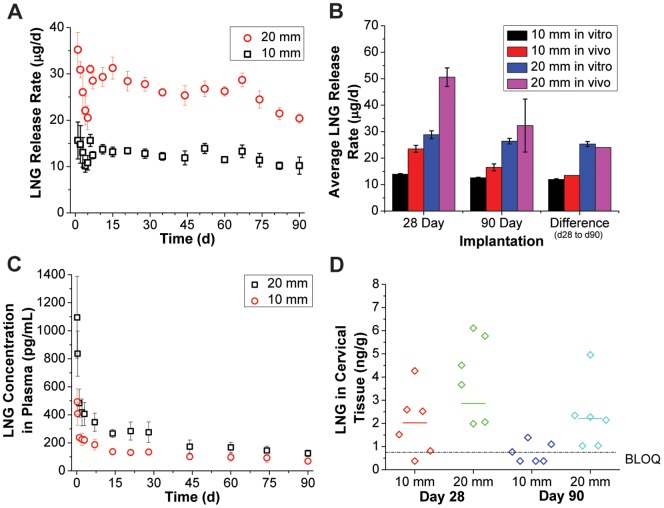
LNG pharmacokinetics in a rabbit model. Pharmacokinetic (PK) testing of end-capped 10 or 20 mm PEU-1/PEU-2 LNG segments in New Zealand white rabbits. (A) Parallel *in vitro* release data for the same LNG segment lot used in the study and (B) comparison with *in vivo* data for the 28 and 90 day study groups. A subtraction of the mean LNG recovery between study groups was performed to directly compare *in vitro* and *in vivo* behavior from day 28 to day 90. (C) Plasma LNG levels measured during the rabbit PK study for 10 and 20 mm LNG segment implantations. (D) Individual and median (bar) LNG levels determined from extractions of cervical tissue. Some samples in the 10 mm study groups were below quantification (BLOQ: <0.750 ng LNG per g tissue). BLOQ data points are graphed as LOQ/2 (0.375 ng/g). *In vitro* data represents N = 5, mean ± SD and *in vivo* data represents N = 6, mean ± SD (except for the *in vivo* “difference” data points, which are subtractions of mean values).

Results from the rabbit pharmacokinetic study are shown in Figure 8. Figure 8A depicts *in vitro* LNG release rates from the same batch used in the rabbit study. On average, 10 and 20 mm segments released 12.6 and 26.4 µg LNG per day *in vitro*, respectively, over the 90 day study. Segments consistently released more LNG *in vivo* than *in vitro* as shown in Figure 8B. Release rates were higher *in vivo* for both the 10 and 20 mm segment groups, however this disparity was diminished in the 90-day group with respect to the 28-day group. In fact, when subtracting the average *in vivo* cumulative LNG release of the 28-day group from the 90-day group, the result was very similar to the time-averaged *in vitro* release rate in both groups (10 mm: 13.5 *in vivo* vs. 12.0 µg/day *in vitro*, 20 mm: 24.0 *in vivo* vs. 25.3 µg/day *in vitro*). This indicates that a higher burst release of LNG occurred *in vivo* than *in vitro*, but that the eventual steady-state regime profile was nearly identical. In all cases, average release rates varied nearly two-fold *in vivo* between the 10 mm and 20 mm segment groups.

LNG plasma results, presented in Figure 8C, strengthened the observation of an additional burst not detected *in vitro*. An approximately 3-fold drop was observed in plasma LNG concentration from the 4 hour to the 7 day time point, while levels remained relatively stable from day 7 to day 28. Plasma levels also dropped markedly from day 28 to day 44 (25% in the 10 mm group and 37% in the 20 mm group), but this may be due to anatomical differences in the two animal groups as plasma was collected from the 28 day group for the first 28 days, and from the 90 day group from day 44 to day 90. Comparatively, plasma levels stabilized from day 44 onward. It is worth noting that test subject body masses increased 14% and 16% over 90 days in the 10 mm and 20 mm test groups, respectively. This may have caused additional attenuation of plasma profiles not expected from the *in vitro* release rate profiles alone. Mean plasma AUC_0–28_ values of 110421 and 218315 pg*hr/mL (ratio: 1.98) and AUC_44–90_ values of 154726 and 261354 pg*hr/mL (ratio: 1.69) were calculated for the 10 mm and 20 mm segment groups, respectively, indicating a dose dependent pharmacokinetic response. This and the agreement of *in vitro* and *in vivo* release rates between day 28 and day 90 suggest that sink conditions exist for LNG *in vivo*, allowing for direct control of LNG pharmacokinetics through polymer selection and device geometry.

Since topical microdose progestin contraception is achieved largely through local effects [45], the pharmacokinetic profile of LNG concentration in the cervical tissue are a potentially more important predictor of eventual device performance. Figure 8D depicts LNG concentrations in cervical tissues collected at day 28 or day 90. Some samples in the 10 mm groups were below the limit of quantification (0.75 ng LNG/g tissue), which hindered a detailed analysis of the data. However, the median value differed two-fold between the 10 and 20 mm groups on day 28 (4.01 vs. 2.02 ng/g), providing further evidence of dose-dependent PK. Also, LNG levels did not differ significantly between tissues taken on day 28 or day 90 (p = 0.10, two-tailed, heteroscedastic t-test), confirming near-zero-order LNG release *in vivo* following the burst.

The rabbit model is useful for early *in vivo* testing of vaginal delivery systems [27], [41], although rabbit vaginal histology varies greatly from that of the human vaginal vault. Furthermore, the exact magnitude of the plasma profile is likely of little significance as LNG pharmacokinetics will vary between species [46]. However, this study confirmed that near zero-order behavior was achieved *in vivo* following the burst period, and that two biologically distinct LNG doses were in fact achieved from the 10 mm and 20 mm length devices.

### Conclusions

Multi-purpose drug delivery systems will need to deliver a physically and chemically diverse set of molecules to target an equally diverse set of indications. Accordingly, there is a need for polymeric materials with a range of properties that are equally chemically diverse. Through successful use of HPEU and PEU in the same device, we have demonstrated that biomedical polyurethanes have potential to fulfill this need for many multi-functional IVR formulations. We have described the design and engineering of a two-segment, dual-reservoir TFV/LNG IVR, with focus on the LNG segment, its incorporation into the full device, and the evaluation of its performance using a combination of *in silico*, *in vitro* and *in vivo* methodologies. Our two-stage, mechanistic drug release model provides an improvement to existing models of cylindrical reservoirs with dissolved drug in the literature and enabled the rational design of the long duration LNG segment. We demonstrated the near-zero-order release of LNG at clinically relevant levels both *in vitro* and *in vivo*. We have also presented design challenges specific to this system, including the prevention of drug diffusion between compartments during long-term storage. Through extension testing we have shown the IVR to be mechanically sound in both the dry and hydrated states. This IVR is the first long-acting multifunctional drug delivery system in clinical development and may prove to be an important advancement for women to control their reproductive health. The co-delivery of two drugs as physicochemically diverse as TFV and LNG and at such different release rates, sustained for 90 days, demonstrates the adaptability of this dual-reservoir polyurethane technology. The platform is easily configured to deliver other drugs [12] and a wide range of doses or combinations for other women's heath challenges.

## Supporting Information

File S1 **Supporting Information.**
(DOCX)
