# Supplementary material for: Engineering a Segmented Dual-Reservoir Polyurethane Intravaginal Ring for Simultaneous Prevention of HIV Transmission and Unwanted Pregnancy
Source: PLoS One. 2014 Mar 5;9(3):e88509. doi: 10.1371/journal.pone.0088509 (PMC3943718; doi:10.1371/journal.pone.0088509)
Supplement: File S1 — Supporting Information. (DOCX) [file pone.0088509.s001.docx]

**Supporting Information**

*Drug Release Model Validation Using Finite-Element Simulation*

In order to assess the validity of the two-stage drug release model for solid reservoirs presented in Equation 11, we also performed graphical finite element simulations of drug release in COMSOL Multiphysics 4. The "Transport of Diluted Species Package" was used to solve Fick's Second Law on a free triangular mesh applied to two-dimensional, circular cross-sections of cylindrical reservoirs (an example is shown in Figure S1A). Identically to Equation 11, a uniform initial condition (*c_0_* = 42 mol/m^3^) was applied to the entire cross-section and a sink boundary (*c(r_o_) = 0*) was applied to the outer edges of the cross-section. Core drug diffusivity was fixed at 5 x 10^-9^ cm^2^/s (not considered in our analytical model as long as it is much larger than the membrane diffusivity). Four different configurations were solved with varying *D, r_o_ and r_i_* (see Table S1). Simulations were carried out for 90 days, and area integrals were taken of the cross-section at each time-point to determine drug concentration per unit length as a function of time. These integrals were converted to LNG mass units and multiplied by a fixed length of 20 mm. Equation 11 was evaluated for each of the configurations listed in Table S1, and daily release rates of both the finite-element simulation and analytical solutions were calculated by subtraction. The two data sets were then compared by computing the coefficient of determination (*R^2^*) for each configuration.

| 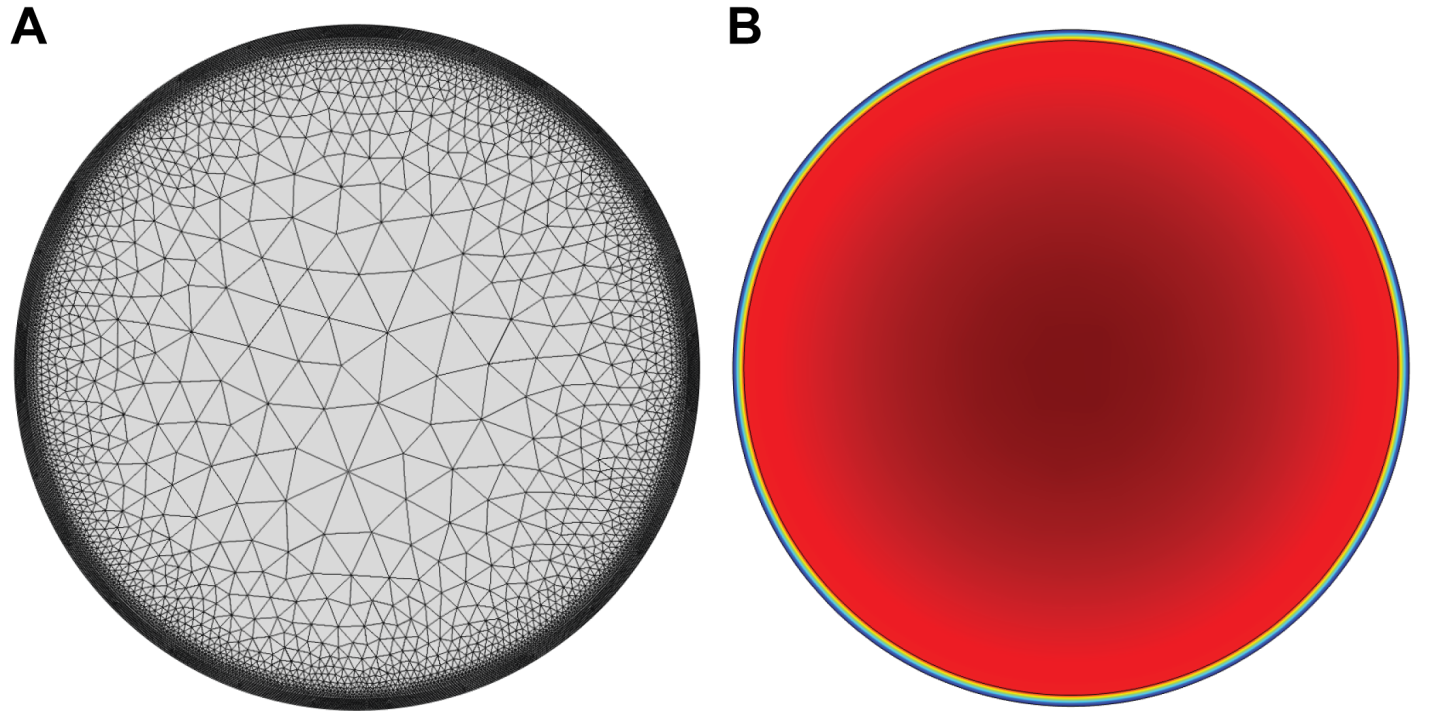 |
| --- |
| **Figure S1:** An example (A) finite-element mesh and (B) final concentration profile result from the finite-element drug release simulation, with highest concentration represented by dark red and lowest concentration by purple. (Configuration 'A' shown, see Table S1) |

| **Table S1:** Values of outer radius (*r_o_*), inner radius (*r_i_*) and drug polymer diffusivity in the outer membrane (*D*) for comparisons of the two-stage drug release model (Eqn. 11) to finite-element diffusion simulations. | | | | | |
| --- | --- | --- | --- | --- | --- |
| Configuration | Outer Diameter (mm) | Wall Thickness (µm) | *r_o_* (mm) | *R_i_* (mm) | *D* (10^-11^ cm^2^/s) |
| A | 5.5 | 90 | 2.75 | 2.66 | 5.0 |
| B | 8.0 | 90 | 4.00 | 3.91 | 1.0 |
| C | 3.0 | 200 | 1.50 | 1.30 | 5.0 |
| D | 5.5 | 200 | 2.75 | 2.55 | 10.0 |

An example concentration profile at the end of the finite-element simulations is shown in Figure S1B, and comparisons of daily release rate profiles from the finite-element simulations and computed using Equation 11 are shown in Figure S2. Equation 11 generally agreed with the simulations with two exceptions. First, the predicted drug release on day 1 was always significantly lower in the simulations compared to Equation 11. Since a concentration discontinuity exists on the outer boundary (*r_o_*) at t=0, there is bound to be some difficulty in accurately predicting the actual day 1 release using a numerical method. Second, two models generally disagreed during the changeover between the burst and steady state regimes of drug release. As noted during the derivation, our model treats this changeover as an instantaneous switch occurring at *(t_b_)*, during which drug fluxes are likely to be underestimated while the concentration profile adjusts to the constraint of the inner boundary at *r_i_*. The models generally agree after this point, except for configuration C, where Equation 11 continued to under-predict the results of the simulation by an average of 18% during the predicted reservoir-stage (from day 19 onward). Based on the configurations that were simulated, the adjustment between the matrix and reservoir concentration profiles likely takes longer for proportionally thicker RCM (higher values of *r_i_/r_o_*). This may be due to the first-term approximation used to predict matrix-stage release (Equation 11a), that is increasingly inaccurate for higher values of cumulative release, and only to be used for the first 20-30% of release (13% is released in the matrix-stage in configuration C). As mentioned in the manuscript, this model will be the most accurate for thinner membranes, and a higher order approximation would need to be used to predict matrix-stage release for proportionally thicker membranes, as well as a re-calculation of the burst duration (*t_b_*). Nonetheless, day 2 to day 90 *R^2^* values were greater than 0.90 for all four configurations, confirming that this is a sufficiently accurate predictor of drug diffusion in cylindrical reservoirs and useful for device design and optimization. Also interesting is that the core does not appear to be completely well mixed in Figure S1B as per our assumption, even with core and membrane diffusivity values that were 100-fold different. However, based on Figure S2A, this does not appear to significantly affect the accuracy of Equation 11 in this setup.

| **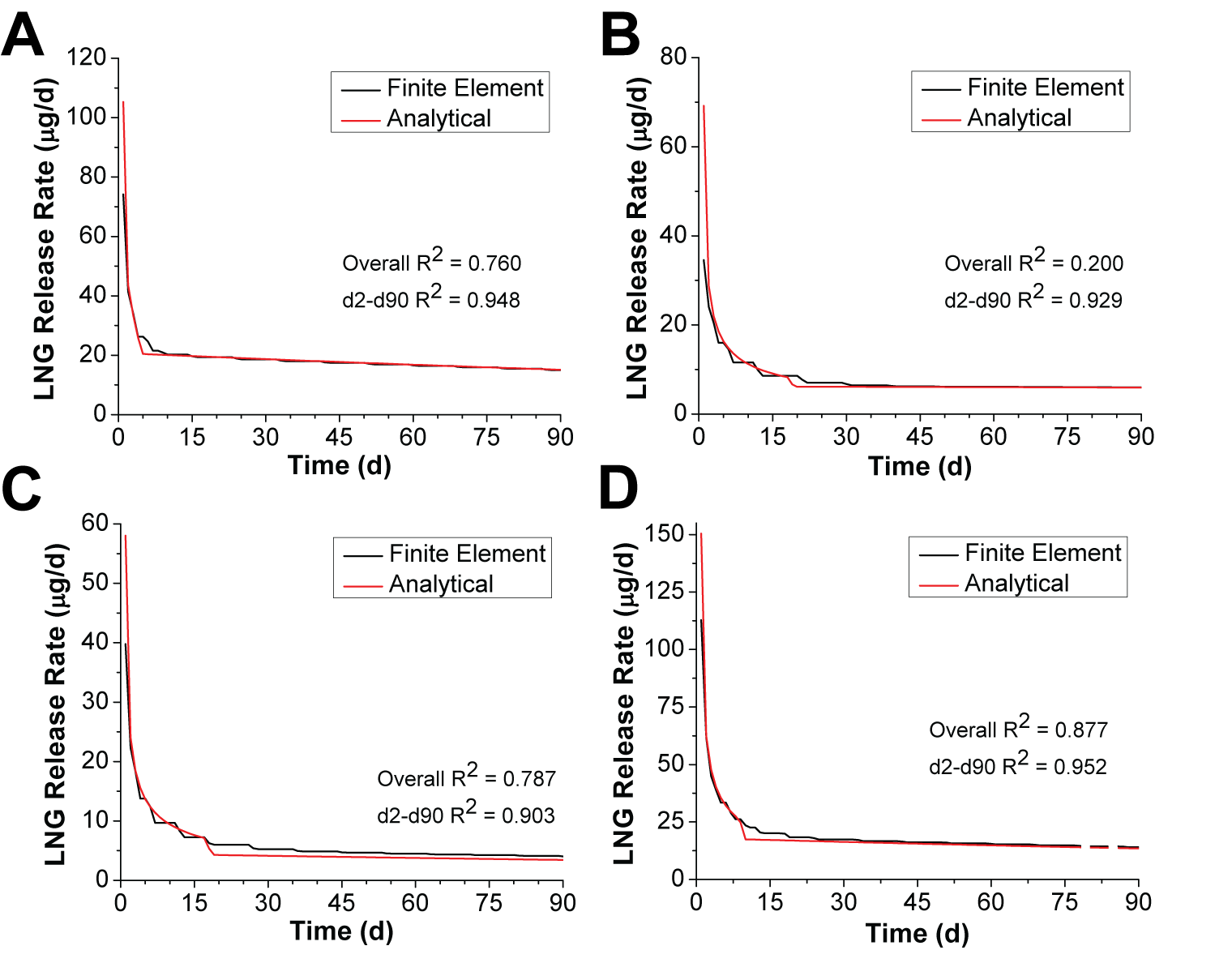** |
| --- |
| **Figure S2:** Comparison of a finite-element diffusion model and our analytical model (Equation 11) for drug release from cylindrical reservoirs. Panels A, B, C and D correspond to the "Configuration" column in Table S1. |

*Gradient HPLC methods for quantification of LNG in in vitro release samples*

Two gradient HPLC methods were used to quantify LNG in *in vitro* release samples. In both cases a 1200 Series HPLC with a quaternary pump and diode array detector (Agilent Technologies, Santa Clara, California) and a 4.6 x 250 mm, 5 µm Zorbax ODS column (also Agilent Technologies) were used. For samples from LNG segment tests a 7-minute gradient of H_2_O and acetonitrile (ACN) was used (Table S2), whereas for samples from full TFV/LNG IVR tests an 18-minute gradient of 0.1% formic acid in H_2_O and 0.1% formic acid in ACN was used (Table S3). In both cases, LNG was detected at 240 nm.

| **Table S2:** HPLC gradient profile for detection of LNG alone in *in vitro* release samples. Line A: H_2_O, Line B: ACN. A flow rate of 1.1 mL/min was applied throughout the method. | | |
| --- | --- | --- |
| Time (min) | % Line A | % Line B |
| 0.0 | 30 | 70 |
| 2.0 | 30 | 70 |
| 2.5 | 20 | 80 |
| 5.0 | 20 | 80 |
| 5.5 | 30 | 70 |
| 7.0 | 30 | 70 |
| **Table S3:** HPLC gradient profile for LNG in TFV/LNG IVR *in vitro* release samples. Line A: 0.1% formic acid in H_2_O, Line B: 0.1% formic acid in ACN. A flow rate of 1.0 mL/min was applied throughout the method. | | |
| Time (min) | % Line A | % Line B |
| 0.0 | 100 | 0 |
| 8.0 | 20 | 80 |
| 13.0 | 20 | 80 |
| 15.0 | 100 | 0 |
| 18.0 | 100 | 0 |

| **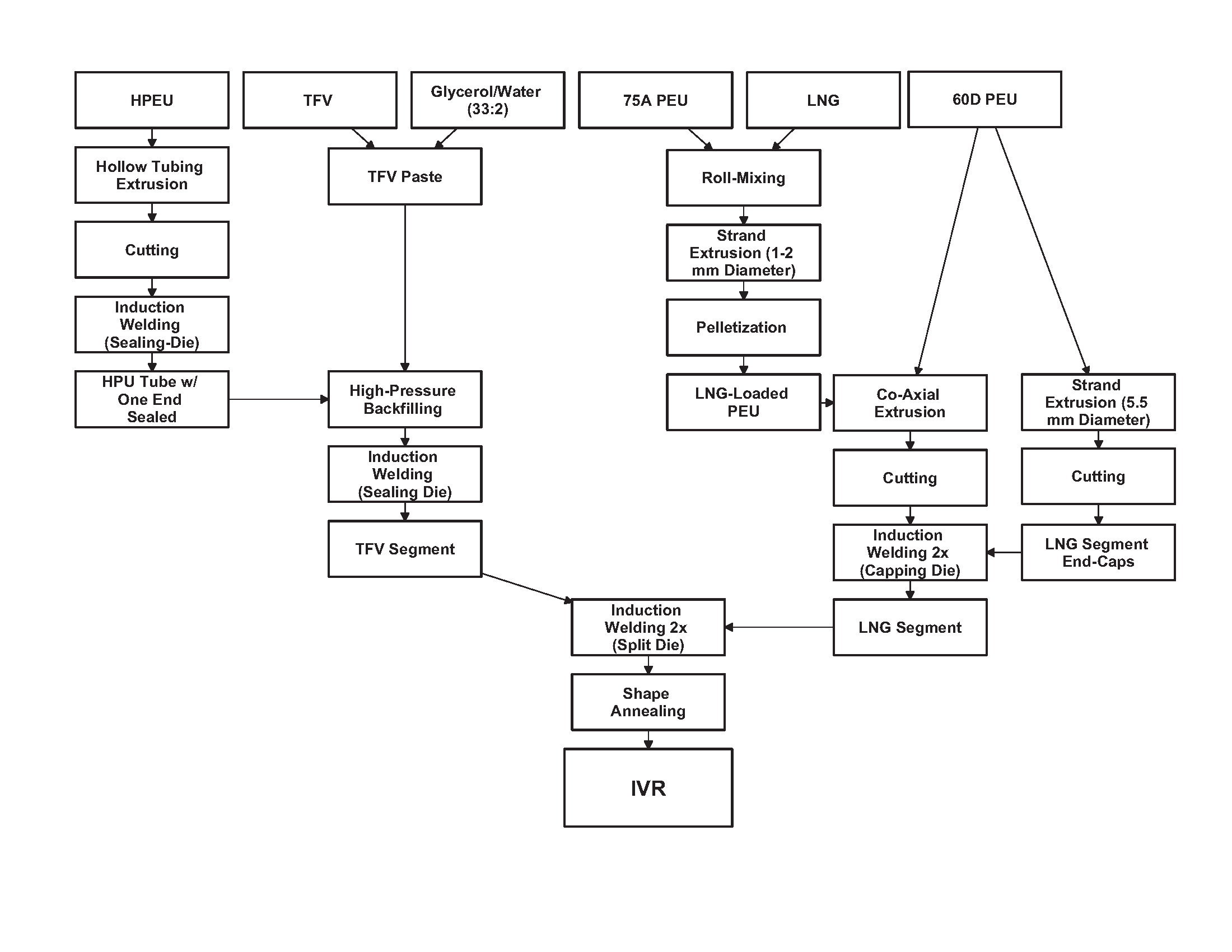** |
| --- |
| **Figure S3:** Manufacturing flow diagram for the TFV/LNG IVR. |
| **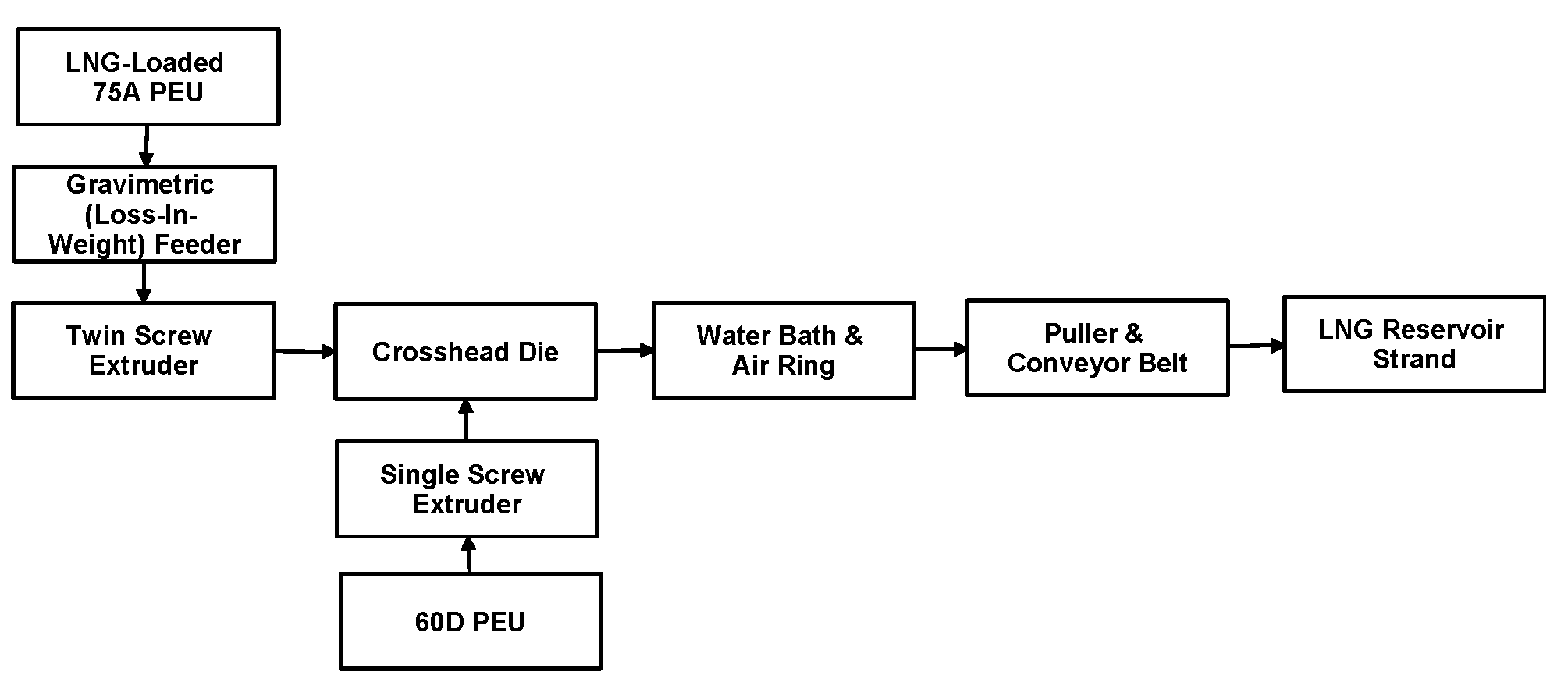** |
| **Figure S4:** Process flow diagram for co-axial extrusion of LNG-loaded PEU strands. |

*Extraction of LNG from rabbit plasma samples and quantification by LC-MS/MS*

LNG was quantified in rabbit plasma samples by LC-MS/MS during the PK study described in the manuscript. Blood samples were first thawed to room temperature, vortexed briefly and centrifuged at 4000 rpm for 10 minutes at 5°C. 250 µL of each sample was added to a 96-well plate. Also to each well were added 400 µL H_2_O and 50 µL of an internal standard containing levonorgestrel-d6 (LNG-d6) (TLC PharmaChem, Inc., Vaughan, Ontario, Canada). In parallel, an Oasis HLB 96-well solid phase extraction (SPE) plate (Waters, Milford, Massachusetts) was conditioned by passing 800 µL MeOH followed by 800 H_2_O, another 800 µL MeOH and finally 800 µL dichloromethane (DCM) under minimal vacuum. Sample mixtures were then transferred to the conditioned SPE plate under minimal vacuum (as needed). 800 µL H_2_O, followed by 800 µL 40:60 MeOH/H_2_O under minimal vacuum was passed through the wells to waste, followed by 5 minutes under high vacuum to dry the SPE beds. 800 µL DCM then was passed through the SPE wells to a 96-well collection plate. High vacuum was again applied to the plate until the SPE beds were visually dry. The DCM eluent was then evaporated under N_2_ at 40°C, and reconstituted with 100 µL 50:50 MeOH/H_2_O. The entire plate was then vortexed for 1 minute, and samples were again centrifuged at 4000 rpm for 5 minutes at 5°C.

LNG was then quantified in the reconstituted extractants using an 1100 Series HPLC (Agilent Technologies) and a API 5000 mass spectrometer (MS) (AB SCIEX, Framingham, Massachusetts). The HPLC was equipped with a chilled autosampler (set to 5°C), as well as two binary solvent pumps and a 6-port switching valve. Two Betasil C_18_ 100 x 2.1 mm, 5 µm columns (Thermo Scientific, West Palm Beach, Florida) were used, one as the loading column and one as the analytical column. Gradients of H_2_O and ACN (both with 0.1% formic acid) were run on both columns. Pump profiles are shown in Table S4 and the column switching profile is shown in Table S5. Positive daughter ions of LNG and LNG-d6 (245.2 and 251.2 m/z) were detected at approximately 4 minutes.

| **Table S4:** HPLC pump profiles for the plasma LC-MS/MS method. Line A: 0.1% formic acid in H_2_O, Line B: 0.1% formic acid in ACN. | | | | | | | |
| --- | --- | --- | --- | --- | --- | --- | --- |
| Pump 1 | | | | Pump 2 | | | |
| Time (min) | Flow Rate (mL/min) | % Line A | % Line B | Time (min) | Flow Rate (mL/min) | % Line A | % Line B |
| 0 | 0.3 | 35 | 65 | 0.0 | 0.6 | 35 | 65 |
| 2.5 | 0.3 | 35 | 65 | 1.0 | 0.6 | 35 | 65 |
| 2.6 | 0.6 | 0 | 100 | 1.1 | 0.3 | 35 | 65 |
| 4.6 | 0.6 | 0 | 100 | 5.4 | 0.3 | 35 | 65 |
| 4.7 | 0.6 | 35 | 65 | 5.5 | 0.6 | 0 | 100 |
| 10.0 | 0.6 | 35 | 65 | 6.5 | 0.6 | 0 | 100 |
|  | | | | 6.6 | 0.6 | 35 | 65 |
|  | | | | 10.0 | 0.6 | 35 | 65 |

| **Table S5:** HPLC switching profile for the plasma analysis method. Position A: Pump 1 - autosampler - Column 1 - waste; Pump 2 - Column 2 - MS, Position B: Pump 2 - Column 1 - Column 2 - MS; Pump 1 - autosampler - waste. | |
| --- | --- |
| Time (min) | Position |
| 0.0 | A |
| 1.4 | B |
| 2.4 | A |

*Extraction of LNG from rabbit cervical tissue samples and quantification by LC-MS/MS*

Rabbit cervical tissue samples were ground with dry ice in a small blade homogenizer to make a finely ground sample. Samples were then stored overnight at -20°C to allow the dry ice to sublime. The ground tissues were then diluted with 80:20 MeOH/H_2_O at 100 mg tissue/mL and sonicated for 5 minutes in an ice water bath. Samples were stored at -80°C until analysis could be performed. Samples were again thawed, vortexed and centrifuged at 4500 rpm and 4°C for 10 minutes. The supernatants were transferred to fresh containers prior to aliquoting into 96-well filter plates. To each well, 600 µL ACN, 50 µL of LNG-d6 internal standard and 250 µL of sample were added. Plates were allowed to stand for approximately 5 minutes before applying vacuum and collecting in a 96-well collection plate. The extractants were evaporated and reconstituted, vortexed and centrifuged as described for plasma extractants above prior to analyzing by LC-MS/MS.

For analysis of tissue extractants, the same LC-MS/MS instrument as above was used except a single-pump, single-column set-up was employed. A 7-minute gradient of 0.1% formic acid in H_2_O and 0.1% formic acid in ACN was run through a Betasil C_18_ 100 x 2.1 mm, 5 µm column. The pump profile is described in Table S6. The same positive daughter ions as above, (245.2 and 251.2) were detected for LNG and LNG-d6 at approximately 2.2 minutes.

| **Table S6:** HPLC gradient profile for LNG detection in cervical tissue using LC-MS/MS. Line A: 0.1% formic acid in H_2_O, Line B: 0.1% formic acid in ACN. A flow rate of 0.3 mL/min was applied throughout the method. | | |
| --- | --- | --- |
| Time (min) | % Line A | % Line B |
| 0.0 | 38 | 62 |
| 2.1 | 38 | 62 |
| 2.2 | 5 | 95 |
| 3.7 | 5 | 95 |
| 3.8 | 38 | 62 |
| 7.0 | 38 | 62 |
